# Understanding L2 Demotivation Among Chinese Tertiary EFL Learners From an Activity Theory Perspective

**DOI:** 10.3389/fpsyg.2021.704430

**Published:** 2021-07-01

**Authors:** Chili Li

**Affiliations:** School of Foreign Languages, Hubei University of Technology, Wuhan, China

**Keywords:** L2 demotivation, Chinese tertiary EFL learners, activity theory, formulating mechanism, mediational factors

## Abstract

This study reports on the results of a qualitative inquiry on second language (L2) demotivation among 14 Chinese EFL learners from the perspective of Activity Theory. Semi-structured interviews were applied to collect data. Through the qualitative content analysis approach, this study found that L2 demotivation prevailed among the participants, influenced by an array of mediational factors including subject-mediated, rule-mediated, community-mediated, tool-mediated, and labour-of-division-mediated factors. The findings imply that L2 demotivation results from the contradictory relationship among the above mentioned factors in the activity system and is a socially mediational construct. The findings shed light on the formulating mechanism of L2 demotivation and provide insightful implications for overcoming the detrimental effect of demotivation in the Chinese EFL context and beyond.

## Introduction

Second language (L2) demotivation has been heatedly discussed in the past decades (Dörnyei, 2001). To date, most previous studies have focused on describing demotivation and identifying its demotivators in English as a foreign language (EFL) class (Hu and Cai, 2010; Li, 2013; Moiinvaziri and Razmjoo, 2014; Kim et al., 2019). These studies suggest that L2 demotivation is caused by both internal and external factors. However, few studies have investigated how these factors function to cause demotivation, which thus necessitates a more comprehensive approach to understand the formulation mechanism of L2 demotivation.

English plays a critical role in the Chinese EFL context. The EFL education in China is supposed to equip the learners with a good command of English proficiency and communicative competence. To this end, Chinese EFL learners start to learn English in the third year at primary school, for 3 years in middle and high school, respectively. At University, students are required to study College English in the first four semesters (Guo et al., 2020). English bears paramount importance to the learners for a number of pragmatic reasons, namely, to pass the high-stake English tests such as the National English Matriculation, College English Test Band 4 (CET-4), to get a good job, and others (Gao et al., 2004).

However, EFL learners in Chinese universities, particularly in non-key local universities are often found to be demotivated. Among them, a belief of uselessness to learn English prevails (Cheng, 2015). Accordingly, they show little interest in class and thus barely engage themselves into English learning. Some of them express that they see little connexion between what they learn from the College English class and the study of their later professional courses (Cai, 2017). It has hence become increasingly important for EFL teachers and educational policy makers to sensitise themselves with learners' experiences when aiming to sustain their L2 motivation against the background of China's initiatives to integrate itself into the globalised world. In order to address the aforementioned theoretical and practical issues, the present study thus aims to explore the formulating mechanism of L2 demotivation among Chinese tertiary EFL learners.

## Literature Review

### Research on L2 Demotivation

Demotivation is initially conceptualised as the external factors that may lead to a decrease in the learner's willingness or continuous behaviours in learning the second or foreign language (Dörnyei, 2001). This conceptualisation does not take the internal factors into account. Demotivation is later interpreted as a decrease in motivation either directly negatively caused by the external factors, or indirectly caused by the detrimental effect of internal factors due to the undesirable influence of the external ones (Falout et al., 2009; Li, 2013).

The earliest attempt to identifying demotivators reveals the following common demotivators: (1) teacher factors, (2) undesirable teaching environment, (3) decreasing confidence, (4) negative attitude towards the target language, (5) the target language as a compulsory subject, (6) interference of anther foreign language under learning, (7) negative attitudes towards the native country associated with the target language, (8) attitudes towards peers around, and (9) textbooks and teaching materials (Dörnyei, 2001).

Scholars from Japan (Weda, 2018; Kikuchi, 2019), Korea (Kim and Kim, 2015; Song and Kim, 2017), and Iran (Moiinvaziri and Razmjoo, 2014) are the most active in exploring L2 demotivation in Asian EFL context. The demotivators found are mainly at teacher, situational, and learner levels.

The primary demotivator reported is related to teachers and their teaching. Teacher personality and teacher-student relationship are found to exert great influence upon learners' demotivation (Weda, 2018; Kikuchi, 2019). Other teacher-related factors such as inappropriate teacher behaviour in class (Kikuchi, 2009), teaching competence and teaching style (Song and Kim, 2017; Kim et al., 2019), teaching methods and stability of teaching faculty (Kikuchi, 2017) are found important in causing demotivation to learn English among the Asian EFL learners.

Situational factors are also important demotivators such as English as a compulsory course (Kim and Kim, 2017), grammar-translation teaching mode (Kikuchi, 2009), non-communicative teaching approach, unsupportive learning environment (Kim and Kim, 2017), negative influence of examinations (Kim and Kim, 2015), the torturing memorization of vocabulary learning, and unfavourable textbook/reference book-related issues and other undesirable facilities (Sakai and Kikuchi, 2009).

Another important demotivator identified pertains to learners. The increasing gap in English proficiency among the learners would lead to decrease in motivation for English learning (Kikuchi, 2019). Low self-esteem, lack of confidence, failure experiences and intrinsic interest are detrimental in motivating the learners (Moiinvaziri and Razmjoo, 2014). Learners' negative perception towards English-speaking countries would also lead to demovation (Kim and Kim, 2015).

Lack of given social value in English learning (Moiinvaziri and Razmjoo, 2014) and excessive social expectation of English proficiency (Song and Kim, 2017) would increase EFL learners' pressure and exert negative influence on their motivation to learn English. Demotivation seems to be discursively constructed in the constant interaction between the individual and the social context (Rashidi et al., 2014).

However, there is a paucity of research on L2 demotivation in China. The limited number of existing research partly confirms the demotivators as identified outside China. The causes responsible for Chinese EFL learners' demotivation are at personal, situational and social levels (Hu and Cai, 2010; Zhou and Wang, 2012; Li, 2013; Li et al., 2020).

The aforementioned review indicates that previous studies mainly adopt a multi-sourced approach, holding that a multitude of demotivating sources lead to the emergence of demotivation. This approach has identified multiple sources of demotivation at personal, situational and socio-cultural levels and deepens our understanding of L2 demotivation. However, most of them still focus on identifying individual demotivators but fail to show the interactional process of these factors that might have worked together to cause demotivation in a particular social context (Li, 2015). Therefore, the formulating mechanism of demotivation is to be further explored. The Activity Theory which integrates the personal and socio-cultural conditions into accounting for the L2 developmental process is thus suggested to accommodate this shortcoming (Kim and Kim, 2013; Li, 2015).

### Demotivation From an Activity Theory Perspective

#### Activity Theory

Activity Theory stems from the Vygotskyan socio-cultural theory (Vygotsky, 1978). It incorporates the key concepts of mediation and contradiction. Mediation refers to the indirect relationship established between the external stimulus and the individuals' approbation of various mediational resources (Cole, 1976). It holds that the realisation of human activity comes from the interaction between the individuals and the social world. This interaction is actualized by means of such mediational tools as learning discourse, cultural artefacts and material conditions, and important social agents (Lantolf, 2000; Gao, 2010).

Activity theory aims to account for the cognitive development of human beings, which is composed of seven core concepts, namely, subject, object, tool, rule, community, labour of division, and outcome (Engeström, 1987). Subject includes individual or groups of learners and agency involving subject's motivation, willingness, and beliefs. Object is the anticipated target of the activity. Tools are the devices that humans use to act on the physical world (Vygotsky, 1978). Rule relates to the system and norm to be complied by the communities of the activities. Community is composed of multiple individuals who share the common general objectives. Labour of division refers to the duties and power relations among the members of the community within the activity system.

Contradiction is the conflicting relationship among the components of an activity system (Kuutti, 1996). The components of the activity system change with the change of the external environment, which might lead to contradiction among these components. Contradiction is a severe but important difficulty which could not be solved by individual behaviours alone, but by the cooperation among the components which could promote the emergence of new activity (Engeström, 1987). Contradiction is solved when an object is successfully transferred into a new anticipated outcome (Basharina, 2007) and would further promote the change and reconstruction of the interaction among the components of the activity system in its execution.

#### A Proposed Framework for L2 Demotivation From Activity Theory Perspective

Based on the aforementioned analysis, demotivation is defined as a socially mediated phenomenon that the realisation of the significance of language learning is impeded or slowed down by the contradiction among the components of the activity system to which the learners are engaged (Li, 2015). It emerges from the contradiction among the components of the activity system. This contradiction would exert negative influences on the effort for the learner to participate in the learning activity system, which would slow down or impede the realisation of the meaningfulness of the activity concerned to the learner (Kim and Kim, 2013).

**Figure 1:**
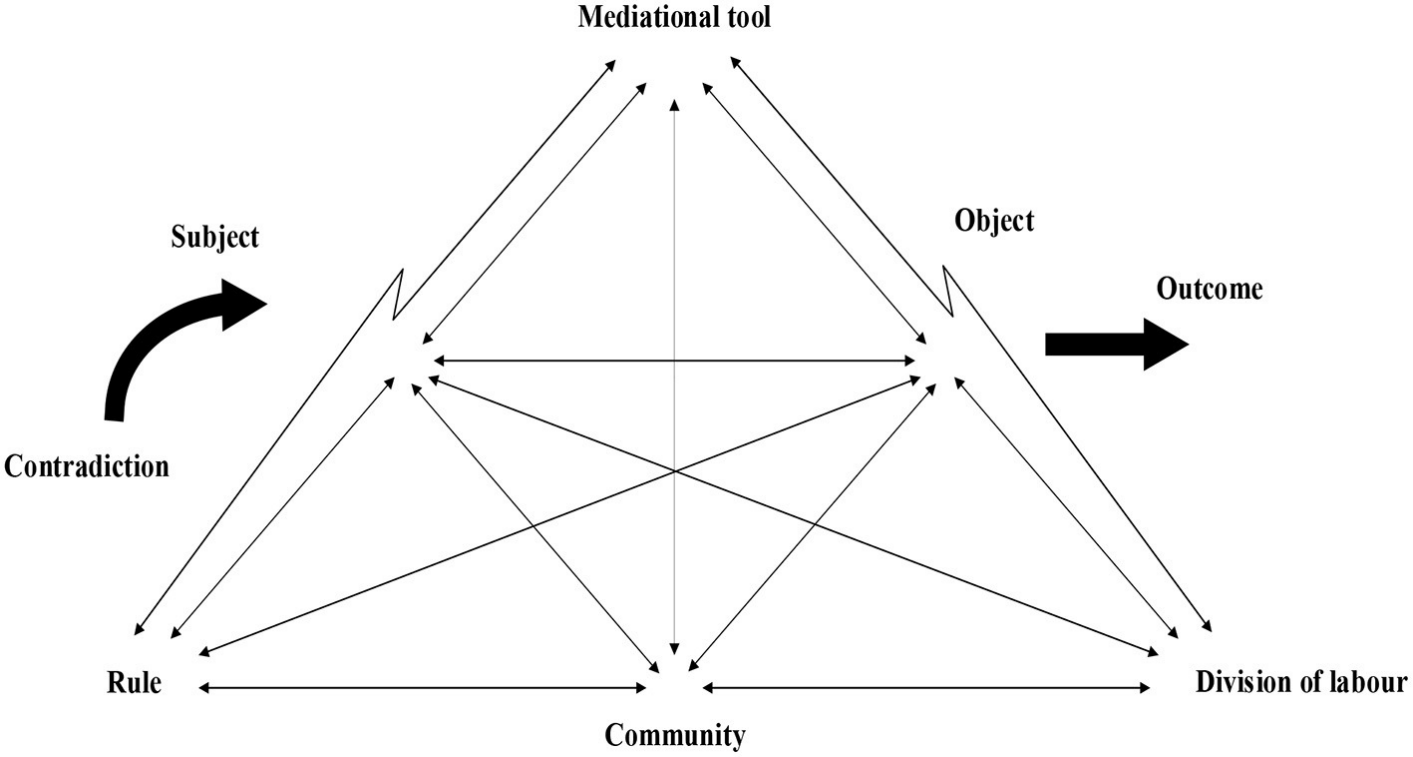
A proposed analytical framework of demotivation.

Framed on previous discussion, an analytical framework for L2 demotivation is proposed (Figure 1). This framework takes L2 demotivation as a complex, multiple-dimensional, and socially mediated process (Rashidi et al., 2014). Its activity system is composed of subject, mediational tools, object, and division of labour, community, rule, and contradiction. Subject refers to learner agency such as will, beliefs about language learning, interest, confidence, learning strategies and others. Object is the target language the learners are learning. Mediational tools include artefacts and material conditions like learning tasks, assignments, textbooks, and facilities. These three parts consist of the fundamental elements of learning activity system.

Learners would apply some mediational tools to learn the language. This learning process does not nestle in a vacuum, but takes place in a certain particular socio-cultural context. This socio-cultural context encompasses rule, community and division of labour. Rule is associated with learning of the language items, exams and achievement. Community includes teachers, students, parents and other family members, as well as socio-cultural background. Division of labour associates with teacher-learner relationship, and peer relationship. The socio-cultural context would exert profound influence upon the language learning valence and beliefs among the learners, and thus make explicit the significance of the learning of the target language to the learners.

The language learning activity is not only mediated by the tools, rules, community and division of labour, but also influenced by learner-related factors. When contradiction emerges from the interaction of these factors, it would either facilitate or block the smooth progression of language learning activity. This proposed analytical framework illustrates the formulation mechanism of demotivation, highlighting the mediation of tools, and explicating the social disposition of learning behaviour from the subjects to the learning outcomes. Demotivation is not only a cognitive behaviour, but a social activity situated in a particular socio-cultural context, resulting from the interplay between individual cognition and contextual conditions.

## Research Design

### Research Questions

1. Do the Chinese Tertiary EFL learners experience demotivation in their English learning at a local technological University in China?
2. What socio-cultural factors might account for the demotivation in learning English among the participants?

### Participants

The population involved in this semi-structured study is composed of 14 Year-3 undergraduate students, composed of ten male students and four female students, respectively. They were pseudonymously named as Adam, Catherine, Donald, Harry, Jack, Jane, Jason, Joe, John, Mike, Rachel, Robin, Smith, and Tracy, respectively. Of these interviewees, one majors in Biology, five in Mechanical Engineering, three in Pharmaceutical Engineering, one in Information Management, three in Electrical Engineering, and one in Civil Engineering. Only two of the interviewees were from metropolitan cities like Wuhan, while the remaining from rural and town areas. With regard to their self-scaled interest in English language learning, only two of them expressed a strong interest, one with moderate interest, and eleven showed no interest. In terms of their English language proficiency, eleven passed the National College English Test Band 4 (CET-4), while two of them failed. The average scores are 459.75 out of the 710 full marks.

The interviewees were invited from a local key technological University located at Wuhan, a metropolitan city of Central China. The University belongs to the first-tier group in the National College Entrance Admission system, which means that the students enrolled by the University have higher marks in the National College Entrance Examination than those admitted by the second—and third-tier universities. It offers its students modules of College English mainly focusing on intensive English reading and listening in the first four semesters of college education. Similar to other local technological universities in China who are currently undergoing a transformational period to be application-oriented institutions of higher education, the University encounters a number of challenges such as over-sized class, reduced teaching hour of College English courses, position of College English course to be orientated at the National College English Test Band-4 instead of the students' needs for English competence for profession knowledge study and real-life communication (Cai, 2012, 2017; Wen, 2015). As a result, students at these universities are often found to be demotivated in the study of College English (Li et al., 2020).

### Interview Protocol

The instrument for the present study is a self-designed interview protocol based on such previous studies as Li (2013), and Zhou and Wang (2012). The instrument is composed of two parts. Part 1 inquires the participants' background information including name, gender, major, grade, self-perceived interest in English learning, English scores obtained in the College English Test Band 4 (CET-4) and hometown. Part 2 investigates the interviewees' demotivators from the perspective of Activity Theory. This part mainly aims to explore the socio-cultural factors that might have caused demotivation in learning English among the interviewees, namely their interest in learning English, the reasons for them to lack interest in learning English (subject- and tool-related factors), their perceived influences of teachers and peers in their English learning experiences (labour-of-division-related factor), their understanding of English learning at local technological University (community-related factor), and their perception about the washback of the reform of the National English Matriculation upon their English learning (rule-related factor).

### Data Collection

The semi-structured interviews were administered in the first week of the Fall semester. The time point was selected out of the following reasons: the interviewees just sat the CET-4 test and their final exam, respectively, in June, from which they might have developed a reasonable understanding of their English language learning experiences. This would help them better express themselves when being interviewed. Besides, upon the new semester, they have got their results of the CET-4 test, which would provide us information on their English language proficiency. Each interview was executed in Chinese and lasted no more than 30 minutes. Every interview was audio-recorded.

### Data Analysis

The collected data regarding the interviewees' self-perceived interest were first counted in order to identify their demotivation to learn English (Research Question 1). Then, qualitative analysis approach (Dörnyei, 2007) was adopted to interpret the results as regards the interviewees' demotivation (Research Question 2). The transcribed data were processed by means of ATLAS.ti and were read and re-read repeatedly for the sake of identifying the possible themes. Guided by the analytical framework, the data texts were initially coded focusing on the mediational sources at levels of object, rule, community, artefacts, labour of division, etc. When the initial coding was completed, the texts were further examined repeatedly so as to identify the commonalities of the emerging themes (Further examples about the mediating factors under community, subject, labour of division, rule, and tool would be given in **Table 2** in section *Socio-Cultural Factors Leading to Demotivation Among the Participants*). To guarantee the coding reliability, one third of the same transcript was re-coded by another researcher in applied linguistics 3 months after the initial coding had been established. The author discussed with the researcher for any discrepancy in the coding. The inter-coder reliability coefficient was 0.925, suggesting a high reliability (Miles and Huberman, 1994).

## Findings

### The Participants' Experience of Demotivation

Table 1 reports the participants' self-scaled level of interest in English learning. There are 11 (78.57%) respondents who scaled their passion for English learning to be at a low level, 1 (7.14%) of them at a medium level, and only 2 (14.29%) of them at a high level. That is to say, over 85% of the surveyed participants are less motivated. This result is echoed in the interviews in which all the interviewees expressed that they ever experienced moments they were reluctant to learn English. This finding also confirms a number of previous studies (e.g., Sakai and Kikuchi, 2009; Hu and Cai, 2010). It could be concluded that demotivation to learn English presents itself among the University students, which indicates demotivation of the technological University students holds true of other groups of University students.

**Table 1:** The participants' self-scaled level of interest in English learning.

**Passion for English learning**	**High**	**Medium**	**Low**	**Total**
	2 (14.29%)	1 (7.14%)	11 (78.57%)	14

### Socio-Cultural Factors Leading to Demotivation Among the Participants

The analysis of the interview data reveals that L2 demotivation among the participants was mediated by the contradiction of the following factors (Table 2), namely, tool-mediated factors, subject-mediated factors, rule-mediated factors, community-mediated factors, and labour-of-division-mediated factors.

**Table 2:** Socio-cultural factors identified in the interviews.

**No**.	**First-level factor**	**Second-level factor**	**Frequency**
1	Community	English language learning valence	11
		Disadvantageous status of English at technological universities	9
2	Subject	Lack of intrinsic interest	17
		Reduced self-confidence	17
		Lack of English learning competence	7
		Unclear English learning goals	4
		Lack of effective learning strategies	4
3	Labour of division	Negative influence of peers	15
		Teacher-learner relationship	32
4	Rule	Exam-oriented teaching mode	8
		Negative influence of Gaokao reform	7
5	Tool	Inadequate teaching facilities	8
		Less interesting topics of contents, tasks and assignments	24
		Over-sized English class	5

#### Tool-Mediated Factors

Several tools were found to have possibly influenced the interviewees' demotivation such as inadequate teaching facilities, less interesting topics of contents, tasks and assignments, and over-sized English class. Less interesting contents of the learning materials, tasks and assignments (24 mentions) were acknowledged as an important demotivator. Just as Interviewee Robin said, “…, *the topics in my English textbook are very boring. I just do not like them*.” Similar opinions also come from Interviewee Harry, “*Many topics of the content in our textbook are out of date, which are not so interesting nowadays*.” Learners, if overloaded would be less likely to experience the joy of English learning, but become more anxious and even demotivated in learning English (Li and Qian, 2018; Kang, 2019).

Other factors such as inadequate teaching facilities (8 mentions), and over-sized English class (5 mentions) are considered to demotivate the students. For example, “*Too large class size will influence the quality of teaching. It is very noisy in a large class to learn English”* (Interviewee Smith). College English classroom is characterised by over-sized class in Chinese universities, local technological universities in particular. A large number of those local universities fail to provide desirable facilities for College English education and have to adopt large-sized class teaching. The large class restricts teacher-student interaction in class and might thus undermine the students' motivation to learn English (Li, 2015).

#### Subject-Mediated Factors

The interview data reveal a number of subject-mediated factors including lack of intrinsic interest, reduced self-confidence, inadequate English learning competence, unclear English learning goal, and lack of effective learning strategies that might have lead to demotivation among the participants.

Lack of intrinsic interest (17 mentions) is mentioned the most by the interviewees, indicating that lack of interest is the primary reason for demotivation among technological students. This could be illustrated by Interviewee Harry, “*I had no interest in English, and this is the reason why I choose Engineering as my major in the National College Entrance Examination. It is thus a big challenge for me to still have English class in my University education*.” This result echoes Hu and Cai (2010). Similarly, reduced self-confidence is another major subject-mediated demotivator (17 mentions). As Interviewee John commented, “*I really tried my best to learn English. However, it really hurt each time I saw the miserable marks in exam*.”

There were 7 mentions in relation to the interviewees' inadequate English learning competence. Interviewee Jason had it: “*Learning English is a burden for me. I really think I do not have that kind of ability to learn it well*.” Some students like Interviewee Jason might think that they cannot learn English well because of lacking the competence of learning English. Because of low English learning competence, some students may not be good at learning language no matter how hard they try. Lack of self-efficacy would make learners easy to give up when confronting difficulties (Li, 2016).

Besides, lack of effective learning strategies (4 mentions) would make learners at a loss in their English learning. What they know is only rote learning. As a consequence, it is very ineffective for them and only makes them resistant to learning English, and eventually causes demotivation among them. As reflected by Interviewee Jack, “*I have no idea what strategies to use to learn English*.”

In addition, unclear goal for English learning (4 mentions) is also mentioned as an important demotivator. Interviewee Jack mentioned, “*I just want to pass the exam. I am at a loss if not for exams*.” Unclear goal of learning English would make learners unable to experience the joy of learning English, except for dealing with the routines of the English exams (Hu and Cai, 2010).

#### Rule-Mediated Factors

The analysis of the interview data suggests that exam-oriented teaching mode and negative influence of National English Matriculation Reform are the two rule-mediated factors that might have caused the learners' demotivation. Exam-oriented teaching mode is reported to be an important demotivator (8 mentions). “*It is very boring to learn English only for the CET-4*” (Interviewee Jack). Most local technological universities prioritise the pass rate of CET-4. This thus makes English learning meaningless to the students but for exams (Kang, 2019). Under the pressure of pursuing CET-4 pass rate, English teachers usually dominate the class, while students become used to listening to teachers' lectures. This grammar-focused and exam-oriented class paid much attention to grammar, drills and recitation, which seemed to be demotivating the students (Li et al., 2020).

Negative influence of National English Matriculation (Gaokao) Reform was reported to be another important demotivator (7 mentions). For example, Interviewee Jack mentioned, “*the National English Matriculation is said to reduce the total scores for the subject of English, which would reduce its importance in the National College Entrance Examination*.” This quote suggests that the misunderstanding of the National English Matriculation Reform seemed to have exerted influences upon the attitudes of Chinese EFL learners towards English learning. This might mislead the public that English would be de-vitalized. Therefore, these stakeholders might come to pay less attention to English education which in turn undermines their motivation for English learning (Cheng, 2015).

#### Community-Mediated Factors

The interviews show that the learners' demotivation was influenced by such community-mediated factors as social value of English learning (11 mentions). “*I barely have any opportunity to use English in my daily life. So, I could really not get a reasonable and convincing reason to study English hard*” (Interviewee Harry). This may be explained by the origin of these interviewees. Most of the respondents were from central-western underdeveloped areas. Living in these less-developed areas they have few opportunities to use English in their future life. These areas had very low degree of internationalisation, where English exerted limited influences upon the life of the locals (Luo, 2015). Therefore, these reasons might have constrained the participants from developing a rational understanding of the social value of English learning, which then further caused demotivation (Lamb, 2013).

The interviews also reveal that the disadvantageous status of English discipline in technological universities (9 mentions) is another community-mediated demotivator. This result has not yet been reported in previous studies. Just like Interviewee Smith said, “*I feel that English at our school does not receive due attention, maybe because this is a technological University where English has no value but for passing CET-4*.” This is related to the marginalisation of English in technological universities. Most technological universities in China are cutting the in-class teaching hours of English courses (Yi and Jiang, 2013). English courses at these universities pay less attention to the students' needs for professional studies (Ma, 2012). The disconnection between the students' needs for profession knowledge and the position of College English results in undesirable efficiency of College English teaching. This situation is deteriorated by the lack of effective transition between College English course and students' professional study, which makes them increasingly unsatisfactory at College English (Qiao, 2012).

#### Labour-Of-Division-Mediated Factors

Labour-of-division-mediated factors include teacher-student relationship and negative influence of peers. According to the interviews, the intense teacher-student relationship was mentioned 32 times that might have caused demotivation in their past English language learning experience. “*My English teacher barely communicates with us and thus I become less enthusiastic about her class*” (Interviewee Adam) and “*My English teacher is so severe that she seldom smiles in class. What is more, she often sighs and gets angry easily*” (Interviewee Smith). Teacher's personality and the teacher-student relationship would possibly influence, or even jeopardise the students' English learning motivation (Li et al., 2020).

Peer influence is another important demotivator (15 mentions). Some participants are likely to be influenced by peers around them. For example, Interviewee Joe seemed to be affected by roommates. “*Every time my roommates study English, I think I should study it as well*.” Likewise, Interviewee Jack's demotivation would emerge with his roommates' undesirable performance. “*I would be embarrassed to study English if my roommates all are playing PC games*” (Interviewee Jack). The competition atmosphere between peers would also exert pressure upon the learners, which might hazard their confidence and thus reduce their motivation to learn English. “*Since I entered University, I have found many classmates around me are very good at English, which gives me a lot of pressure*” (Interviewee Tracy).

## Discussion

This study has identified five mediational factors relating to subject, rule, community labour of division, and artefacts that might have caused demotivation among the participants. These factors display the following characteristics:

Firstly, subject-mediated factors seem to be the most influential. This result shows the similarities in L2 demotivation among the technological University students with other populations of English learners and validates the important role of internal factors in L2 demotivation (Li, 2016). This study identified that lack of effective learning strategies are one of the internal demotivators, which were not frequently reported in other studies and thus enriched the existing literature. This finding might be explained as follows: College English class is often found to be teacher-dominated and pays little attention to the cultivation of students' strategic awareness. Therefore, this lack of effective learning strategies would possibly cause anxiety and frustration when learners fail to choose appropriate strategies to solve language learning difficulties.

The second remarkable feature is related to teachers (community-mediated factor) and teacher-student relationship (labour-of-division-mediated factor). This result confirms previous findings reported by Dörnyei (2001) and Sakai and Kikuchi (2009). Traditional teacher-dominated and exam-oriented teaching mode fails to take into account the individual differences of the learners and thus fails to satisfy their needs in English learning (Li, 2016).

A third salient feature is the exam-related rule-mediated factor. For those participants, to pass CET-4 is a huge challenge. This pressure would be amplified as the result of teacher factors and of exam-oriented education like over-loaded learning tasks and exercises. If inappropriately handled, the stress would lead to weariness in English learning as a result of which demotivation would emerge (Kim and Seo, 2012).

**Figure 2:**
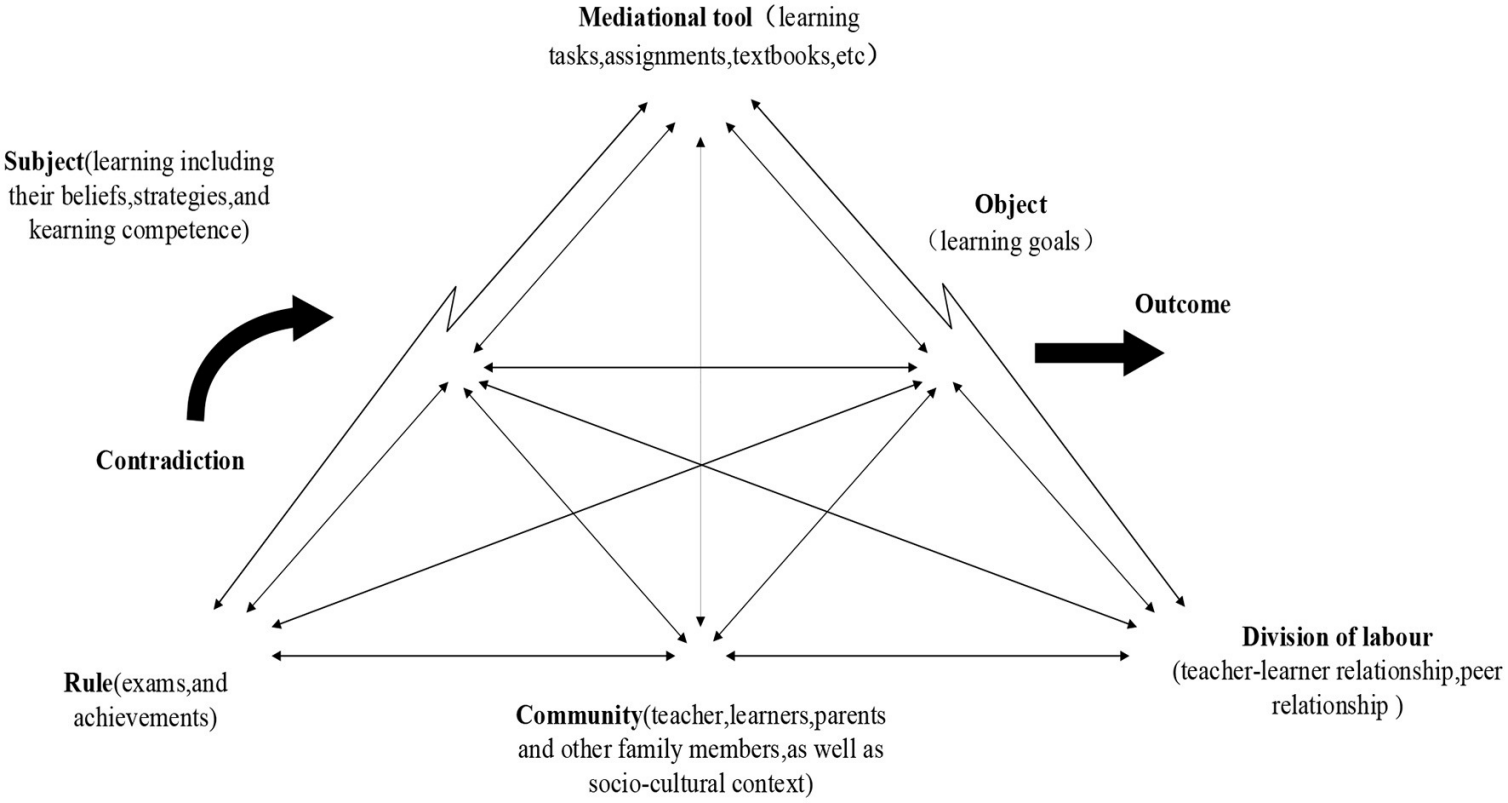
Demotivators from the activity theory perspective.

This study found that the relationship between L2 demotivation and the socio-cultural factors could be interpreted by means of the proposed analytical framework from activity theory (Li, 2015). L2 demotivation is not individualistic behaviour, but a socio-culturally mediated phenomenon and a result of collective behaviour in the learning activity system. When contradiction appears in the components of the activity system, L2 demotivation would thus come out. The mediational factors of contradiction identified in the present study illustrate the mechanism of L2 demotivation among the technological University students (Figure 2).

This study found that subject-mediated factors such as lack of effective learning strategies, reduced confidence and lack of intrinsic interest are influential determinants in causing demotivation. Activity theory holds that learning goal has a promoting effect when subject exerts influence upon object (Engeström, 2001). When learners hold a negative perception of English learning, their motivation to learn English would be hazardously influenced (Kim, 2011). The participants' perception of the low English learning valence might be associated with their family background. Most of the participants come from the Midwestern part of China and have limited opportunities to contact English. English language has no influential effect on their future employment and personal development. These reasons could thus account for their perception of the English language valence which might have caused their demotivation to learn English (Lamb, 2013).

The learners' reduced confidence and lack of intrinsic interest might be caused by their failure experience in English learning. This experience might in turn be related to the difficulties in their English learning, such as bulk of learning tasks such as exam-oriented exercises (artefacts-mediated factor). These may be also related to their lack of effective English language learning strategies (subject-mediated factors).

When subjected to criticism from teachers and competition against peers (labour of division-mediated factors), the learners, being short of immature cognitive competence, become incapable of regulating these pressures. As a result, they tend to be easily frustrated at their English learning. At the same time, their lack of effective learning strategies might result from the teacher-dominated and exam-oriented teaching mode (community-mediated factor) which ignores the cultivation of the students' strategic competence.

The above analysis reveals that the components of the demotivation system are interrelated to each other and exert interwoven effects upon the contradiction while the subjects apply various mediational tools to realise the outcome of the object. This further illustrates the formulating mechanism of demotivation in English language learning.

## Conclusion

The present study explored the L2 demotivation among a group of Chinese EFL learners at a technological University from the activity theory perspective. It has found that most of the participants ever experienced demotivation in their English learning. Their demotivation is mediated by the tool-mediated, the subject-mediated, the rule-mediated, the community-mediated, and the division-of-labour-mediated factors. This study contributes to the present literature in that the disadvantageous status of English in technological universities and the negative influence of National English Matriculation Reform are the factors particular to the EFL learners at local technological universities. It bridges the gap that little attention is paid to L2 demotivation among Chinese EFL learners from the activity theory perspective. It provides empirical evidence to the feasibility of applying Activity Theory into analysing L2 demotivation, and offers a new theoretical perspective for L2 demotivation research. This study sheds light on the interactional relationship among personal, situational and social factors which might have aligned to lead to demotivation of Chinese EFL learners.

This study has some pedagogical implications. Local technological universities are advised to bridge the gap between the position of College English and the needs of the students in their professional study. Teacher development programmes such as motivational strategies are suggested to be included to empower EFL teachers (Zeynali et al., 2019). Students should be guided to develop a reasonable understanding of English language learning value at technological universities. Teachers might also help the learners to clarify their misunderstanding of the reform of English in the National College Entrance Examination, help them set clear goal (Hamada, 2014), and guide them to foster reasonable attitudes towards exam scores in EFL learning. As for peer influence on learners' demotivation, they might be trained how to interact with peers in EFL learning so as to create a positive image of peers and peer competition (Tanaka, 2017).

This study suffers from some limitations for its single data sources from semi-interviews and its small number of sample population. Further research is suggested to enrich study with larger population from a wider scope and apply both quantitative and qualitative methods to seek empirical evidence for the analytical model proposed in the present study.

## Data Availability Statement

The raw data supporting the conclusions of this article will be made available by the authors, without undue reservation.

## Author Contributions

The author confirms being the sole contributor of this work and has approved it for publication.

## Conflict of Interest

The author declares that the research was conducted in the absence of any commercial or financial relationships that could be construed as a potential conflict of interest.

## References

[B1] Basharina O. K. (2007). Activity theory perspective on student-reported contradictions in international telecollabouration. Lang. Learn. Technol. 11, 82–103. 10.1016/10125/44105

[B2] Cai J. (2012). Re-analysis of the goal of College English teaching in the perspective of globalisation. For. Lang. Teach. 29, 5–8. 10.13458/j.cnki.flatt.000502

[B3] Cai J. (2017). “Debates around the orientation of TEFL in Chinese tertiary education,” in Innovation in Language Learning and Teaching: The Case of China, eds H. Reinders, D. Nunan, and B. Zou (London: Palgrave), 115–154. 10.1057/978-1-137-60092-9_6

[B4] Cheng X. T. (2015). The butterfly effect of the reform on national matriculation english test. J. For. Lang. 38, 28–29.

[B5] Cole M. (1976). “Foreword,” in Cognitive Development: Its Cultural and Social Foundations, ed A. R. Luria (Cambridge: Harvard University Press), xi–xvi.

[B6] Dörnyei Z. (2001). Teaching and Researching Motivation. Harlow: Longman. 10.1075/hop.5.mot1

[B7] Dörnyei Z. (2007). Research Methods in Applied Linguistics: Quantitative, Qualitative and Mixed Methodologies. Oxford: Oxford University Press.

[B8] Engeström Y. (1987). Learning by Expanding: An Activity Theoretical Approach to Developmental Research. Helsinki: Orienta-Konsultit.

[B9] Engeström Y. (2001). From Teams to Knots: Activity-Theoretical Studies of Collaboration and Learning at Work. Cambridge: Cambridge University Press.

[B10] Falout J. Elwood J. Hood M. (2009). Demotivation: affective states and learning outcomes. System 37, 403–417. 10.1016/j.system.2009.03.004

[B11] Gao X. S. (2010). Strategic Language Learning: The Roles of Agency and Context. London: Multilingual Matters. 10.21832/9781847692450

[B12] Gao Y. Zhao Y. Cheng Y. Zhou Y. (2004). Motivation types of Chinese University undergraduates. Asian J. English Lang. Teach. 14, 45–64.

[B13] Guo Y. Xu J. F. Xu X. F. (2020). An investigation into EFL learners' motivational dynamics during a group communicative task: a classroom-based case study. System 89, 1–15. 10.1016/j.system.2020.102214

[B14] Hamada Y. (2014). Japanese high school EFL learners' perceptions of strategies for preventing demotivation. Asian EFL J. 75, 4–20.

[B15] Hu W. X. Cai J. T. (2010). Construction of model of demotivation in english learning. For. Lang. Educ. 31, 41–49. 10.16362/j.cnki.cn61-1023/h.2010.03.020

[B16] Kang S. G. (2019). Seeking to relieve demotivation for Korean college students learning English. J. Pan Pac. Assoc. Appl. Linguist. 23, 21–36. 10.25256/PAAL.23.2.2

[B17] Kikuchi K. (2009). Listening to our learners' voices: what demotivates Japanese high school students? Lang. Teach. Res. 13, 453–471. 10.1177/1362168809341520

[B18] Kikuchi K. (2017). Reexamining demotivators and motivators: a longitudinal study of Japanese freshmen's dynamic system in an EFL context. Innov. Lang. Learn. Teach. 11, 128–145. 10.1080/17501229.2015.1076427

[B19] Kikuchi K. (2019). Motivation and demotivation over two years: a case study of English language learners in Japan. Stud. Sec. Lang. Learn. Teach. 9, 157–175. 10.14746/ssllt.2019.9.1.7

[B20] Kim T. Y. (2011). Korean elementary school students' English learning demotivation: a comparative survey study. Asia Pac. Educ. Rev. 12, 1–11. 10.1007/s12564-010-9113-1

[B21] Kim T. Y. Kim M. S. (2017). Demotivators and remotivation strategies in L2 learning: a case study of Korean EFL students. For. Lang. Educ. 24, 45–74. 10.15334/FLE.2017.24.2.45

[B22] Kim T. Y. Kim Y. K. (2013). Reconceptualising L2 learning demotivation from a Vygotskian activity theory perspective. English Teach. 68, 141–163. 10.15858/engtea.68.4.201312.141

[B23] Kim T. Y. Kim Y. K. (2015). Elderly Korean Learners' participation in english learning through lifelong education: focusing on motivation and demotivation. Educ. Gerontol. 41, 120–135. 10.1080/03601277.2014.929345

[B24] Kim T. Y. Kim Y. M. Kim J. Y. (2019). Role of resilience in (De)motivation and second language proficiency: cases of korean elementary school students. J. Psycholinguist. Res. 48, 371–389. 10.1007/s10936-018-9609-0 30374803

[B25] Kim T. Y. Seo H. S. (2012). Elementary school students' foreign language learning dennotivation: a mixed methods study of Korean EFL context. Asia Pac. Educ. Res. 21, 160–171.

[B26] Kuutti K. (1996). “Activity theory as a potential framework for human-computer interaction research,” in Context and Consciousness: Activity Theory and Human-Computer Interaction, ed B. A. Nardi (Cambridge, MA: MIT Press), 17–44.

[B27] Lamb M. (2013). ‘Your mum and dad can't teach you': constraints on agency among rural learners of English in the developing world. J. Multiling. Multicult. Dev. 34, 14–29. 10.1080/01434632.2012.697467

[B28] Lantolf J. P. (2000). Second language learning as a mediated process. Lang. Teach. 33, 79–96. 10.1017/S0261444800015329

[B29] Li C. L. (2015). On the theoretical construction of negative impetus in foreign language learning based on the perspective of activity theory. English Teach. 15, 45–50.

[B30] Li C. L. (2016). An empirical study on the demotivators in rural middle school learners' english learning. Basic For. Lang. Educ. 18, 14–17.

[B31] Li C. L. Dou R. Zhang S. (2020). A correlational study on psychological resilience and L2 demotivation among Chinese EFL learners. Rev. Argent. Clín. Psicol. 29, 670–681. 10.24205/03276716.2020.770

[B32] Li C. L. Qian J. H. (2018). Investigating changes in demotivation among Chinese EFL learners from an activity theory perspective. Int. J. English Linguist. 8, 44–53. 10.5539/ijel.v8n1p44

[B33] Li L. (2013). A study on internal factors of Chinese college EFL learners' demotivation. J. PLA Univ. For. Lang. 36, 65–69. 10.1002/722X(2013)02-0065-05

[B34] Luo Z. P. (2015). Chinese Netizens' English language attitudes in the context of college entrance examination reform. J. Tianjin Foreign Stud. Univ. 22, 57–63. 10.08/665X(2015)06-0057-07

[B35] Ma L. W. (2012). The individualized contents of college English teaching in engineering colleges — based on the demand of learners and social workplace for english. J. Jiangxi Normal Univ. 45, 141–144. 10.1000/579(2012)06-0141-04

[B36] Miles M. B. Huberman A. M. (1994). Qualitative Data Analysis: An Expanded Sourcebook. Thousand Oaks, CA: Sage Publications.

[B37] Moiinvaziri M. Razmjoo S. A. (2014). Demotivating factors affecting undergraduate learners of non-english majors studying general english: a case of Iranian EFL context. J. Teach. Lang. Skills 5, 41–61.

[B38] Qiao X. L. (2012). Reform in college English in the light of PETOE. Shandong For. Lang. Teach. 33, 69–74. 10.16482/j.sdwy37-1026.2012.03.018

[B39] Rashidi N. Rahimi M. Alimorad Z. (2014). Iranian University English learners' discursive demotivation construction. Iran. J. Lang. Teach. Res. 2, 35–49.

[B40] Sakai H. Kikuchi K. (2009). An analysis of demotivators in the EFL classroom. System 37, 57–69. 10.1016/j.system.2008.09.005

[B41] Song B. S. Kim T. Y. (2017). The dynamics of demotivation and remotivation among Korean high school EFL students. System 65, 90–103. 10.1016/j.system.2016.12.010

[B42] Tanaka M. (2017). Examining EFL vocabulary learning motivation in a demotivating learning environment. System 65, 130–138. 10.1016/j.system.2017.01.010

[B43] Vygotsky L. S. (1978). Mind in Society. Cambridge: Harvard University Press.

[B44] Weda S. (2018). Demotivational teaching practices in EFL classroom: perceptions of English among Indonesian learners. Asian EFL J. 20, 400–414.

[B45] Wen Q. F. (2015). Developing a theoretical system of production-oriented approach in language teaching. For. Lang. Teach. Res. 47, 547–558. 10.000/0429(2015)04-0547-12

[B46] Yi A. S. Jiang D. C. (2013). College English curriculum reform in engineering colleges under the background of internationalization. J. Chongqing Univ. Technol. 27, 118–121. 10.3969/j.issn.1674-8425(s).2013.12.024

[B47] Zeynali S. Pishghadam R. Fatemi A. H. (2019). Identifying the motivational and demotivational factors influencing students' academic achievements in language education. Learn. Motiv. 68, 1–12. 10.1016/j.lmot.2019.101598

[B48] Zhou C. B. Wang W. B. (2012). Demotivators analysis of Chinese University EFL learners. For. Langu. China 45, 48–55. 10.13564/j.cnki.issn.1672-9382.2012.01.013

